# Responses of plant growth, physiological, gas exchange parameters of super and non-super rice to rhizosphere temperature at the tillering stage

**DOI:** 10.1038/s41598-019-47031-9

**Published:** 2019-07-23

**Authors:** Shuying Li, Haolin Jiang, Jianjun Wang, Yandong Wang, Shenggang Pan, Hua Tian, Meiyang Duan, Shuli Wang, Xiangru Tang, Zhaowen Mo

**Affiliations:** 1 College of Agriculture, South China Agricultural University, Guangzhou, China; 2 College of Agronomy and Biotechnology, China Agricultural University, Beijing, China; 3 Scientific Observing and Experimental Station of Crop Cultivation in South China, Ministry of Agriculture. P. R. China, Guangzhou, 510642 Guangdong, China

**Keywords:** Abiotic, Plant sciences

## Abstract

Temperature is one of the critical factors affecting rice growth and yield production. This study investigated the effects of rhizosphere temperature at the tillering stage on the growth, physiological parameters and gas exchange attributes of two rice cultivars, i.e., the super rice cultivar Yuxiangyouzhan and the non-super rice cultivar Xiangyaxiangzhan, under hydroponic conditions. Three rhizosphere temperature treatments, i.e., a low-rhizosphere-temperature treatment (LT, nutrient solution at 17.5 ± 1.5 °C), an ambient-temperature treatment (AT, nutrient solution at 27.5 ± 1.5 °C), and a high-rhizosphere-temperature treatment (HT, nutrient solution at 37.5 ± 1.5 °C), were applied in this study. The results showed significant differences in most of the rice growth and physiological and gas exchange parameters as a result of cultivar and rhizosphere temperature as well as their interaction. In addition, the marked reduction in total dry weight was positively correlated with a notable reduction in plant morphological parameters, such as the fresh and dry weight of the leaves and stem sheaths, and changes in gas exchange parameters. Moreover, antioxidant reactions were active in response to high and low rhizosphere temperatures, which varied in different plant tissues. These results suggested that the super and non-super rice were sensitive to high and low rhizosphere temperatures, respectively.

## Introduction

Rice is one of the main food crops worldwide and plays an important role in maintaining food security in China^1,2^. Super rice varieties have high yield potential and strong growth, nutrient assimilation and resistance capabilities compared to the non-super rice cultivars^3–6^. With respect to obtaining stable production, plant responses to various adverse environmental stresses can be major factors influencing crop yields^7–9^. Specifically, rice plants are sensitive to adverse temperatures^10–12^. For example, high nighttime temperatures decrease rice yields^13^. Genotypes and growth stages vary in response to extreme temperatures, and extreme temperatures can cause yield and quality declines^14–16^. Therefore, research on the responses of rice genotypes to adverse temperatures is needed to address rice production under extreme temperatures.

Generally, rice yields are significantly reduced by extreme temperatures^17,18^. Grain quality, such as the head rice yield and grain width, can decrease under low- or high-temperature conditions, while chalkiness can increase^11,12^. Warm temperature conditions can lead to high growth rates during plant development^19^. Elevated temperatures result in marked decreases in grain and biomass yields but result in increased in some plant growth parameters, such as tillering number, panicle number, panicle length and harvest indexes^20^. Increased nighttime temperatures decrease the filled grain percentage and yield of rice^21^. Moreover, high temperatures reduce grain yields due to the reduction in yield-related traits and physiological injury under high temperatures^22^. In addition, high temperatures can lead to reductions in photosynthesis, respiration, and physiological parameters, as well as RuBP carboxylase activity^23^. Moreover, increased photosynthesis is associated with the improvement of Rubisco under high temperature^24^. Leaf photosynthesis and transpiration of seedlings increase under high water temperature^25^. Further, a high day/night ratio can result in unfavorable growth, yield and grain quality formation, and compared with high daytime temperature, high nighttime temperature has a strong effect on grain weight and some grain quality parameters^26^. Additionally, grain number per panicle, panicle length, and grain weight were decline in response to cold climates^27^. Rice seed germination and some physiological parameters during seed germination are affected by low temperature^28–30^. Low temperature (19–20 °C) results in a reduction in photosynthetic parameters and affects antioxidant enzyme activity and membrane lipid peroxidation^31^. A previous study suggested that the difference in high air temperature and soil temperature could affect carbohydrate metabolism in plants^32^. Increasing the root-zone temperature has a positive effect on plant development^33^. Root grsowth is affected by various soil temperatures^34^, and root temperature significantly affects root growth and function^35^. Additionally, soil temperature significantly affects root vigor, root proline content and MDA concentration^36^. Soil temperature also affects photosynthesis^37^. Thus, high or low temperature during the day or at night in the air or in the soil as well as high- or low-temperature irrigated water for rice plants could result in different growth responses in rice plants.

Effects of air temperature and root-zone temperature on the yield, photosynthetic parameters and antioxidant enzyme activity of rice have been detected^32,33,35,38^, but studies on the effects of rhizosphere temperature on the growth, physiological attributes and gas exchange parameters of super rice and non-super rice varieties during tillering are still limited. Hence, two rice cultivars, i.e., a super rice (Yuxiangyouzhan) and a non-super rice (Xiangyaxiangzhan) cultivar, were grown under three rhizosphere temperatures levels, i.e., low temperature (LT, 17.5 ± 1.5 °C), ambient temperature (AT, 28 ± 1.0 °C) and high temperature (HT, 38.5 ± 1.5 °C), for the purposes of evaluating the influence of rhizosphere temperature on the growth and physiological response of super and non-super rice varieties and investigating the correlations between the investigated parameters under different rhizosphere temperatures for super rice and non-super rice.

## Results

### Variance analysis

The results of the variance analysis depicted a significant effect on agronomic traits except for leaf fresh weight per area, leaf dry weight per area and the root-shoot ratio in the different cultivars. The SOD activity, POD activity, MDA content, CAT activity in roots, proline content in the leaves and stomatal conductance were markedly affected by the variety. The SOD activity in the roots, MDA content in the stems, intercellular CO_2_ concentration, agronomic traits, SOD activity, POD activity, CAT activity, MDA content, proline content, stomatal conductance, photosynthetic rate and transpiration rate exhibited a striking change in response to the temperature treatments, while plant height, leaf fresh weight per area, root dry weight, and leaf dry weight per area did not.

The interaction of cultivar and temperature also resulted in a significant effect on agronomic traits, SOD activity, POD activity, CAT activity, MDA content, proline content, whereas SPAD values, leaf area, leaf fresh weight, leaf fresh weight per area, root dry weight, stem sheath dry weight, leaf dry weight per area, SOD activity in the roots, MDA activity in the stems and proline content in the roots were not affected (Table 1).

**Table 1:** Variance analysis.

Parameters	C	T	C × T
SPAD	13.20*	15.38**	1.59 ns
Plant height	61.01**	3.48 ns	4.98*
Leaf area	6016.99**	6.84**	1.18 ns
Root fresh weight	67.84**	5.63*	8.72**
Stem and sheath fresh weight	125.15**	10.33**	5.24*
Leaf fresh weight	177.12**	16.73**	2.43 ns
Leaf fresh weight per area	1.17 ns	2.41 ns	0.46 ns
Total fresh weight	186.85**	14.04**	6.09**
Root dry weight	147.43**	1.18 ns	1.92 ns
Stem and sheath dry weight	149.36**	7.30**	3.28 ns
Leaf dry weight	136.12**	7.38**	4.76*
Leaf dry weight per area	0.60 ns	0.06 ns	1.00 ns
Total dry weight	284.32**	5.14*	4.12*
Root-shoot ratio	0.73 ns	26.36**	3.88*
SOD activity in Root	40.13**	0.83 ns	2.52 ns
SOD activity in stem	545.81**	13.78**	5.34*
SOD activity in leaf	669.70**	99.91**	168.81**
POD activity in Root	15.52*	5.71*	11.38**
POD activity in stem	219.85**	102.67**	43.60**
POD activity in leaf	1585.05**	479.25**	1376.59**
CAT activity in Root	7.11 ns	23.84**	6.61*
CAT activity in stem	4.15 ns	5.42*	30.29**
CAT activity in leaf	185.42**	446.75**	519.85**
MDA content in Root	26.88*	22.19**	25.03**
MDA content in stem	71.48**	2.51 ns	2.28 ns
MDA content in leaf	19.04*	101.52**	19.74**
Proline content in Root	0.02 ns	5.22*	3.82 ns
Proline content in stem	9.22 ns	26.00**	14.04**
Proline content in leaf	94.77**	9.28**	6.46*
Intercellular CO2 concentration	12.88 ns	1.11 ns	0.35 ns
Stomatal conduction	28.43*	10.25**	0.14 ns
Photosynthetic rate	9.26 ns	8.12*	1.19 ns
Transpiration rate	12.26 ns	6.44*	0.98 ns

C represents the cultivar; T represents the temperature treatment; C × T represents the interaction between cultivars and treatments. *Significant at *p* < 0.05 according to LSD tests; **Significant at *p* < 0.01 according to LSD tests; ns represents not significant according to LSD tests.

### Effects of temperature treatment on organ dry weight and the root-shoot ratio

For Yuxiangyouzhan, the total dry weight and stem sheath dry weight decreased significantly in the high-temperature treatment, with decreases of 10.55% and 12.89%, respectively, while no significant decrease was observed in the low-temperature treatment. Additionally, all temperature treatments caused a significant reduction in leaf dry weight, with declines of 17.22% and 19.95%, respectively, in the low-temperature treatment and high-temperature treatment for Yuxiangyouzhan; however, a significant increase in the root-shoot ratio, with improvements of 42.09% and 34.99%, respectively, in the low-temperature and high-temperature treatments occurred for Yuxiangyouzhan. For Xiangyaxiangzhan, the total dry weight, stem sheath dry weight and leaf dry weight decreased significantly under the low-temperature treatment, with reductions of 41.06%, 52.09% and 40.68%, respectively, while there were no effects in the high-temperature treatment. Additionally, the low-temperature treatment caused a significant increase in the root-shoot ratio (79.19%). No significant effect on root dry weight or leaf dry weight per area in the temperature treatments for either cultivar was observed (Table 2). Thus, under low temperatures, non-super rice exhibited a pronounced reduction in total dry weight and dry weight of the leaves and stems, and these parameters were not notably affected in super rice except for dry weight. Super rice showed a marked decrease in total dry weight and dry weight of the leaves and stems, although these investigated parameters were not markedly influenced by non-super rice.

**Table 2:** Effects of temperature treatment on organ dry weight and the root-shoot ratio.

Treatment	Root dry weight (g)	Stem and sheath dry weight (g)	Leaf dry weight (g)	Leaf dry weight per area (mg•cm^-2^)	Total dry weight (g)	Root-shoot ratio
Yuxiangyouzhan						
LT	0.22a	0.52ab	0.52b	3.00a	1.25ab	0.21a
AT	0.18a	0.60a	0.62a	2.97ab	1.40a	0.15b
HT	0.19a	0.48b	0.50b	2.69b	1.17b	0.20a
Mean	0.20	0.53	0.55	2.89	1.27	0.19
Xiangyaxiangzhan						
LT	0.08a	0.14b	0.18b	2.87a	0.41b	0.26a
AT	0.09a	0.30a	0.31a	3.03a	0.69a	0.14b
HT	0.11a	0.26a	0.34a	3.39a	0.71a	0.19b
Mean	0.09	0.23	0.27	3.10	0.60	0.20

LT represents low rhizosphere temperature (17.5 ± 1.5 °C); AT represents ambient rhizosphere temperature (28 ± 1.0 °C); HT represents high rhizosphere temperature (38.5 ± 1.5 °C). The different letters in the same column represent significant differences at *p* < 0.05 according to LSD tests.

### Effects of temperature treatment on organ fresh weight

A significant reduction in total fresh weight and leaf fresh weight was detected in the temperature treatment for Yuxiangyouzhan. In addition, stem sheath fresh weight decreased significantly in the high-temperature treatment and had but was not markedly affected in the low-temperature treatment. Rather, leaf fresh weight per area was decreased significantly in the low-temperature treatment and was not affected by the high-temperature treatment. For Xiangyaxiangzhan, the total fresh weight, root fresh weight, stem sheath fresh weight and leaf fresh weight decreased significantly in the low-temperature treatment and were affected by the high temperature. Moreover, the temperature treatments had no effect on leaf fresh weight per area (Table 3). Hence, the low rhizosphere temperature caused a significant reduction in the total fresh weight and fresh weight of the leaves of the two rice cultivars. The fresh weight of the stem sheaths and roots of the non-super rice decreased substantially, while these investigated parameters were not notably influenced by the super rice. Under high rhizosphere temperatures, the super rice exhibited a pronounced reduction in total fresh weight and fresh weight of the leaves and stems; however, these parameters were not notably influenced in the non-super rice.

**Table 3:** Effects of temperature treatment on organ fresh weight.

Treatment	Root fresh weight (g)	Stem and sheath fresh weight (g)	Leaf fresh weight (g)	Leaf fresh weight per area (mg•cm^2^)	Total fresh weight (g)
Yuxiangyouzhan					
LT	1.44a	2.46ab	1.40c	8.13b	5.30b
AT	1.40a	2.87a	2.00a	9.55a	6.27a
HT	1.36a	2.33b	1.67b	9.06ab	5.37b
Mean	1.40	2.55	1.69	8.91	5.65
Xiangyaxiangzhan					
LT	0.26b	0.73b	0.56b	8.77a	1.55b
AT	0.79a	1.73a	0.98a	9.60a	3.49a
HT	0.94a	1.60a	1.05a	10.30a	3.59a
Mean	0.66	1.35	0.86	9.55	2.88

LT represents low rhizosphere temperature (17.5 ± 1.5 °C); AT represents ambient rhizosphere temperature (28 ± 1.0 °C); HT represents high rhizosphere temperature (38.5 ± 1.5 °C). The different letters in the same column represent significant differences at *p* < 0.05 according to LSD tests.

### Effects of temperature treatment on SPAD values, plant height and leaf area

SPAD values decreased significantly in the low-temperature treatment, while there was no effect in the high-temperature treatment in both varieties. For Yuxiangyouzhan, a significant reduction in leaf area was detected in the low-temperature treatment, and no effect was detected in the high-temperature treatment. In addition, plant height was not significantly affected by any temperature treatment. The low-temperature treatment resulted in a significant decrease in plant height and leaf area, while plant height and leaf area showed no significant difference in the high-temperature treatment. Therefore, the SPAD value and leaf area were reduced significantly in the low rhizosphere temperature for super rice and non-super rice, while no marked effect was observed in the high rhizosphere temperature for either rice cultivar (Table 4).

**Table 4:** Effects of temperature treatment on SPAD values, plant height and leaf area.

Treatment	SPAD	Plant height(cm)	Leaf area(cm^2^)
Yuxiangyouzhan			
LT	32.05b	47.32a	172.25b
AT	34.57a	46.93ab	210.91a
HT	34.73a	42.88b	185.73ab
Mean	33.78	45.71	189.63
Xiangyaxiangzhan			
LT	29.78b	33.40b	64.13b
AT	32.87a	40.22a	102.40a
HT	34.95a	37.47ab	105.92a
Mean	32.53	37.03	90.82

LT represents low rhizosphere temperature (17.5 ± 1.5 °C); AT represents ambient rhizosphere temperature (28 ± 1.0 °C); HT represents high rhizosphere temperature (38.5 ± 1.5 °C). The different letters in the same column represent significant differences at *p* < 0.05 according to LSD tests.

### Effects of temperature treatment on SOD activity in the roots, stem sheaths and leaves

**Figure 1:**
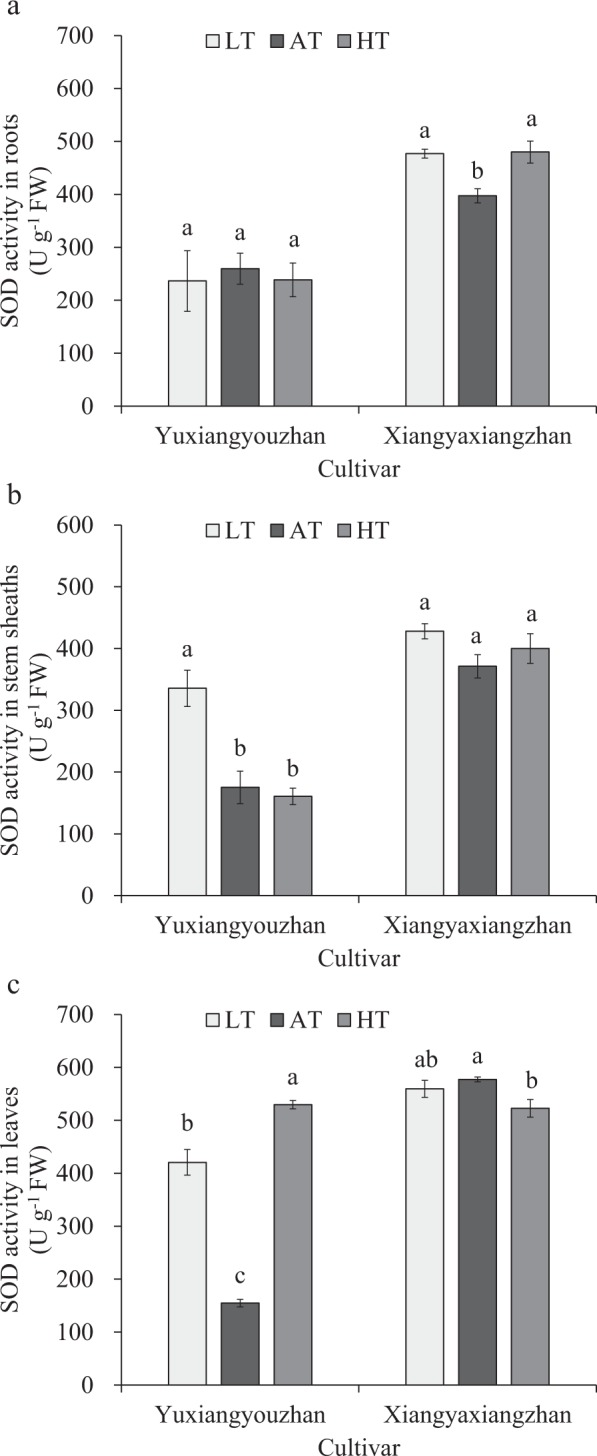
Effects of temperature treatment on SOD activity in the roots, stem sheaths and leaves. (**a**) SOD activity in the roots under three temperature treatments in Yuxiangyouzhan and Xiangyaxiangzhan. (**b**) SOD activity in the stem sheaths under three temperature treatments in Yuxiangyouzhan and Xiangyaxiangzhan. (**c**) SOD activity in the leaves under three temperature treatments in Yuxiangyouzhan and Xiangyaxiangzhan. LT represents low rhizosphere temperature (17.5 ± 1.5 °C); AT represents ambient rhizosphere temperature (28 ± 1.0 °C); HT represents high rhizosphere temperature (38.5 ± 1.5 °C). The vertical bars with different lowercase letters above are significantly different at *p* = 0.05 according to LSD tests. The capped bars represent the SDs.

No significant effect on SOD activity in the roots was found in the temperature treatment for Yuxiangyouzhan. Additionally, the low-temperature treatment caused a significant increase in SOD activity in the stems and leaves. SOD activity in the stems was not significantly affected in the high-temperature treatment, while a significant increase in SOD activity in the leaves was found in Yuxiangyouzhan. The temperature treatment resulted in a significant increase in SOD activity in the roots of Xiangyaxiangzhan but no significant effect on SOD activity in the stems of Xiangyaxiangzhan. SOD activity in leaves decreased significantly in the high-temperature treatment, while there was no difference in the low-temperature treatment. Therefore, under low rhizosphere temperature, SOD activity in the roots increased strongly for non-super rice, while there was no notable effect in super rice. Likewise, SOD activity in the leaves increased substantially under high rhizosphere temperatures for super rice, while there was no marked influence in the same treatment for non-super rice (Fig. 1).

### Effects of temperature treatment on POD activity in the roots, stem sheaths and leaves

**Figure 2:**
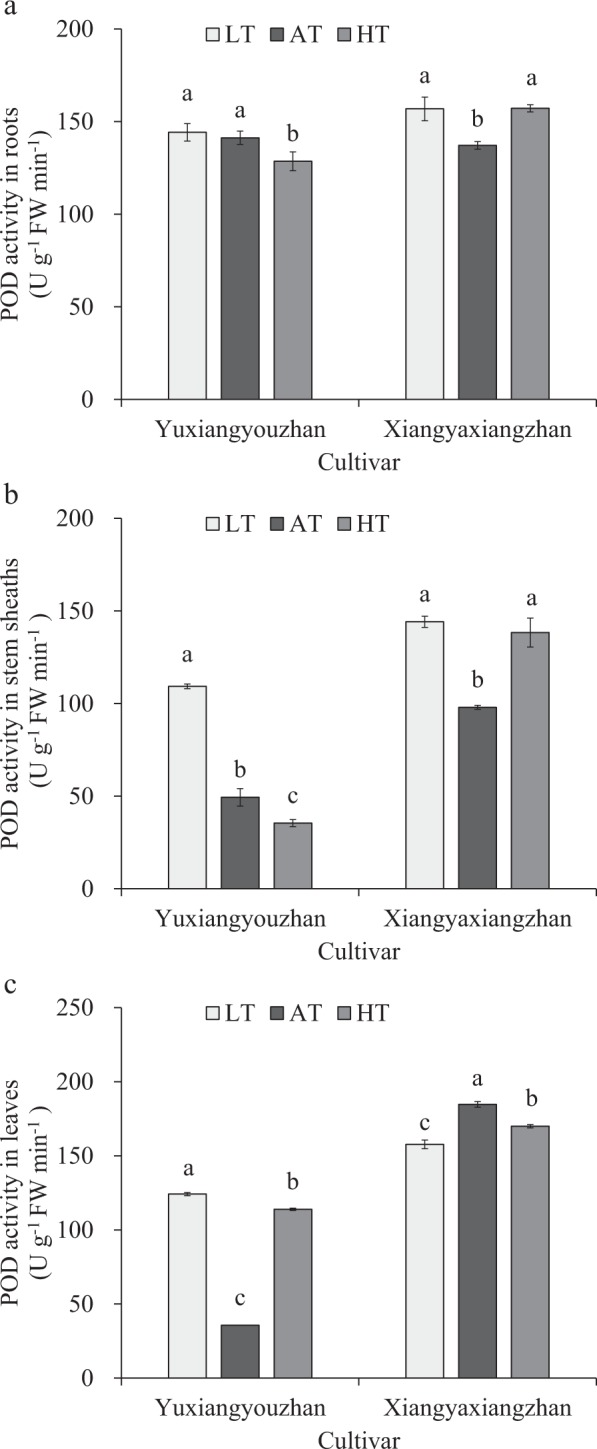
Effects of temperature treatment on POD activity in roots, stem sheaths and leaves. (**a**) POD activity in the roots under three temperature treatments in Yuxiangyouzhan and Xiangyaxiangzhan. (**b**) POD activity in the stem sheaths under three temperature treatments in Yuxiangyouzhan and Xiangyaxiangzhan. (**c**) POD activity in the leaves under three temperature treatments in Yuxiangyouzhan and Xiangyaxiangzhan. LT represents low rhizosphere temperature (17.5 ± 1.5 °C); AT represents ambient rhizosphere temperature (28 ± 1.0 °C); HT represents high rhizosphere temperature (38.5 ± 1.5 °C). The vertical bars with different lowercase letters above are significantly different at *p* = 0.05 according to LSD tests. The capped bars represent the SDs.

The POD activity in different organs between the different cultivars varied. For Yuxiangyouzhan, the low-temperature treatment resulted in a significant increase in the stems and leaves, while no significant difference was detected in the roots. In addition, the high-temperature treatment caused a significant reduction in the roots and stems, and a significant increase was detected in the leaves at high temperature. It indicated that the low rhizosphere temperature resulted in a significantly increased POD activity in the stems of the two rice cultivars; however, the POD activity in the leaves was inhibited substantially at low temperatures in non-super rice but was promoted strikingly at low temperatures in super rice. The high rhizosphere temperatures caused a significant increase in POD activity in the leaves and a marked reduction in POD activity in the stems and roots of super rice, while contrasting results were detected in the non-super rice (Fig. 2).

### Effects of temperature treatment on CAT activity in the roots, stem sheaths and leaves

**Figure 3:**
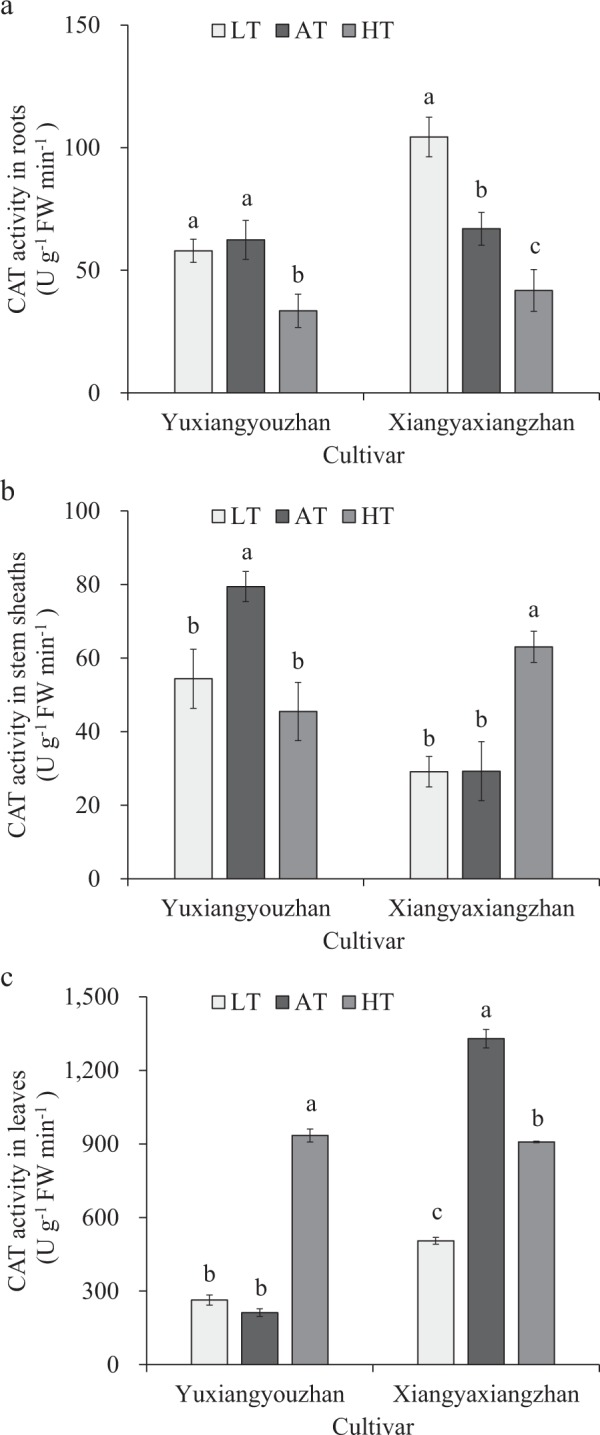
Effects of temperature treatment on CAT activity in the roots, stem sheaths and leaves. (**a**) CAT activity in the roots under three temperature treatments in Yuxiangyouzhan and Xiangyaxiangzhan. (**b**) CAT activity in the stem sheaths under three temperature treatments in Yuxiangyouzhan and Xiangyaxiangzhan. (**c**) CAT activity in the leaves under three temperature treatments in Yuxiangyouzhan and Xiangyaxiangzhan. LT represents low rhizosphere temperature (17.5 ± 1.5 °C); AT represents ambient rhizosphere temperature (28 ± 1.0 °C); HT represents high rhizosphere temperature (38.5 ± 1.5 °C). The vertical bars with different lowercase letters above are significantly different at *p* = 0.05 according to LSD tests. The capped bars represent the SDs.

The high-temperature treatment caused a significant reduction in CAT activity in the roots and stems of Yuxiangyouzhan, while there was a significant increase in the leaves. In addition, the low-temperature treatment resulted in a significant decrease in CAT activity in the stems of Yuxiangyouzhan, while there was no significant effect in the roots or leaves. For Xiangyaxiangzhan, CAT activity increased significantly in the roots in the low-temperature treatment but decreased significantly in the high-temperature treatment. In the stems, no significant effect in CAT activity was detected in the low-temperature treatment, while a significant increase in CAT activity was detected in the high-temperature treatment. In the leaves, CAT activity decreased significantly in all the temperature treatments. Hence, low rhizosphere temperature caused a significant inhibition of CAT activity in the leaves of non-super rice and a pronounced increase in the roots, while no marked effect was observed in super rice. The high rhizosphere temperature resulted in a notable reduction in CAT activity in the roots of the two rice cultivars. The CAT activity in the leaves increased significantly at high rhizosphere temperatures in the super rice, while the CAT activity in stems was reduced strongly at high rhizosphere temperatures in the non-super rice. The results of super rice under the high rhizosphere temperature were opposite those of non-super rice under the high rhizosphere temperature (Fig. 3).

### Effects of temperature treatment on MDA contents in the roots, stem sheaths and leaves

**Figure 4:**
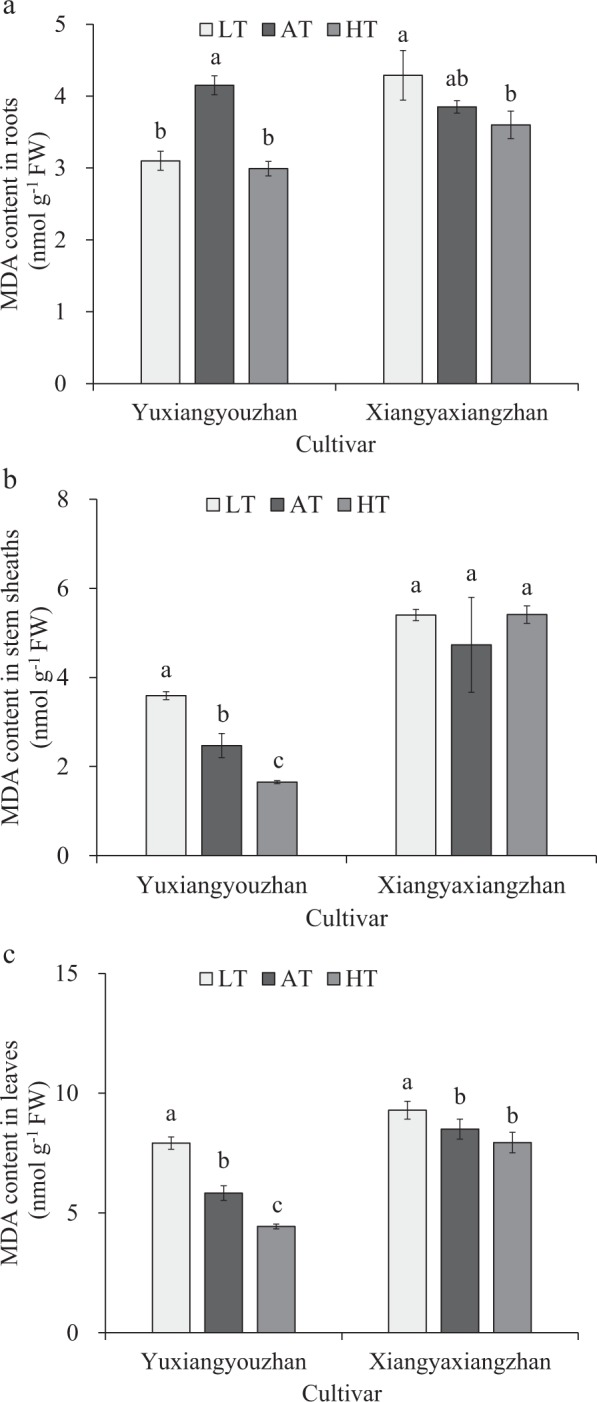
Effects of temperature treatment on the MDA content in the roots, stem sheaths and leaves. (**a**) MDA content in the roots under three temperature treatments in Yuxiangyouzhan and Xiangyaxiangzhan. (**b**) MDA content in the stem sheaths under three temperature treatments in Yuxiangyouzhan and Xiangyaxiangzhan. (**c)** MDA content in the leaves under three temperature treatments in Yuxiangyouzhan and Xiangyaxiangzhan. LT represents low rhizosphere temperature (17.5 ± 1.5 °C); AT represents ambient rhizosphere temperature (28 ± 1.0 °C); HT represents high rhizosphere temperature (38.5 ± 1.5 °C). The vertical bars with different lowercase letters above are significantly different at *p* = 0.05 according to LSD tests. The capped bars represent the SDs.

The MDA content in the roots, stems and leaves was measured. The low-temperature treatment caused a significant increase in the stems and leaves but a significant reduction in the roots of Yuxiangyouzhan. Additionally, the high-temperature treatment caused a significant decrease in the roots, stems and leaves of Yuxiangyouzhan. For Xiangyaxiangzhan, no significant effect was found in the roots and stems in any of the temperature treatments. Additionally, the MDA content increased significantly at low temperatures in the leaves, and there was no significant difference in the high-temperature treatment in the leaves. Consequently, the low rhizosphere temperature resulted in a pronounced increase in MDA contents in the leaves of the two rice cultivars, while the MDA content in the roots of the non-super rice was not markedly affected by the low rhizosphere temperature. The MDA content in the roots of the super rice decreased significantly in response to the low rhizosphere temperature. The MDA content in the roots was reduced substantially by high rhizosphere temperature for the two rice cultivars. However, high rhizosphere temperature caused a marked decrease in MDA content in the leaves and stems of the super rice, with no notable effect on the MDA content in leaves and stems of the non-super rice (Fig. 4).

### Effects of temperature treatment on proline contents in roots, stem sheaths and leaves

**Figure 5:**
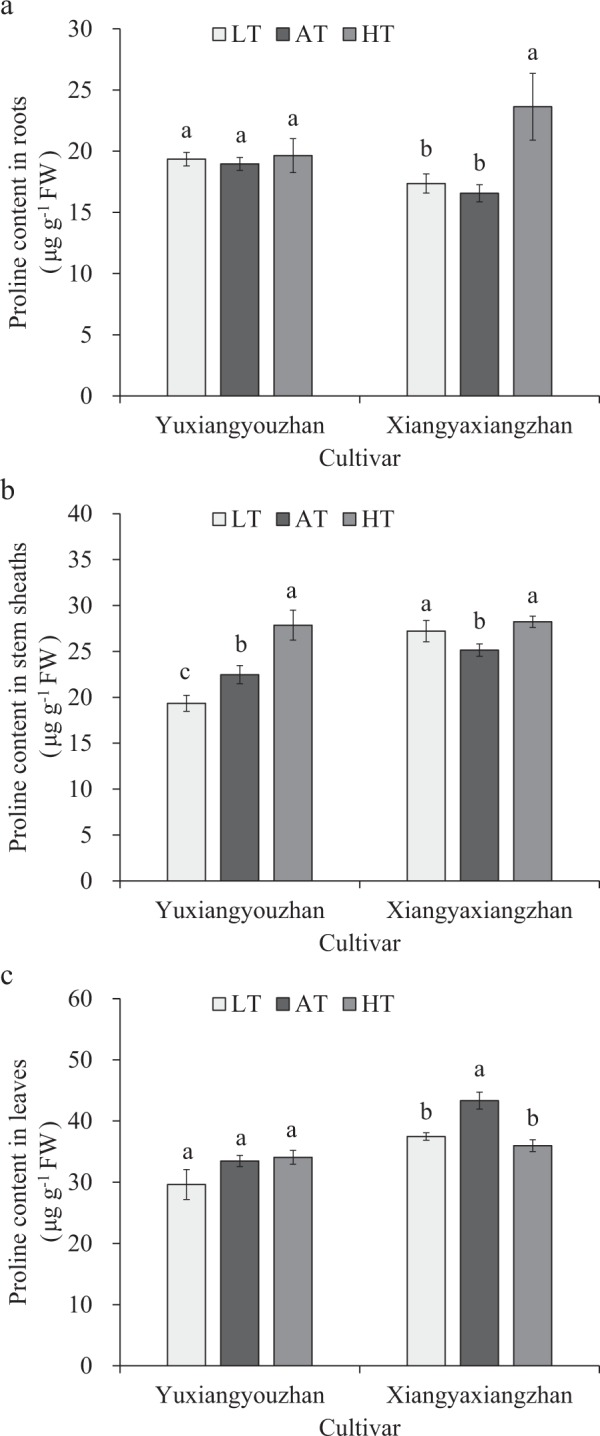
Effects of temperature treatment on the proline content in the roots, stem sheaths and leaves. (**a**) Proline content in the roots under three temperature treatments in Yuxiangyouzhan and Xiangyaxiangzhan. (**b**) Proline content in the stem sheaths under three temperature treatments in Yuxiangyouzhan and Xiangyaxiangzhan. (**c**) Proline content in the leaves under three temperature treatments in Yuxiangyouzhan and Xiangyaxiangzhan. LT represents low rhizosphere temperature (17.5 ± 1.5 °C); AT represents ambient rhizosphere temperature (28 ± 1.0 °C); HT represents high rhizosphere temperature (38.5 ± 1.5 °C). The vertical bars with different lowercase letters above are significantly different at *p* = 0.05 according to LSD tests. The capped bars represent the SDs.

The proline content in the stem sheaths declined sharply in the low-temperature treatment for Yuxiangyouzhan, while it significantly increased in the high-temperature treatment. Additionally, for Xiangyaxiangzhan, the low temperature caused a notable increase in proline content in the stem sheaths and a striking decrease in proline content in the leaves. Significant increases in proline contents in the roots and stem sheaths were detected at high temperatures, while a notable decrease in proline content was detected in the leaves of Xiangyaxiangzhan. However, regardless of the temperature treatment, the proline content was no markedly affected in the roots and leaves of Yuxiangyouzhan. In addition, the proline content in the roots of Xiangyaxiangzhan was not affected by the low-temperature treatment. Thus, the low rhizosphere temperature resulted in a significant decrease in the proline content in the roots of non-super rice, while the opposite effect was observed for the proline content in the roots of super rice. The proline content in the stems significantly increased at high rhizosphere temperatures in the two rice cultivars. Moreover, under high rhizosphere temperature, the non-super rice appeared to have a significant increase in proline content, while the super rice cultivar appeared to have no influence on the proline content (Fig. 5).

### Effects of temperature treatment on the intercellular CO_2_ concentration, stomatal conductance, net photosynthetic rate and transpiration rate

**Figure 6:**
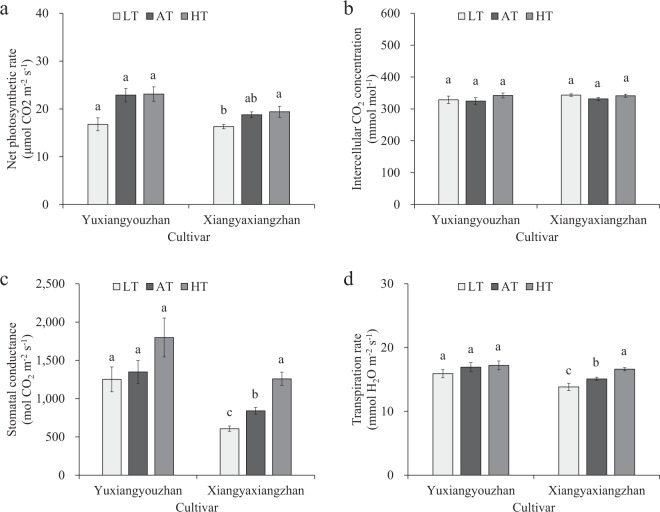
Effects of temperature treatments on the net photosynthetic rate, intercellular CO_2_ concentration, stomatal conductance and transpiration rate of the leaves. (**a**) Net photosynthetic rate under three temperature treatments in Yuxiangyouzhan and Xiangyaxiangzhan. (**b**) Intercellular CO_2_ concentration under three temperature treatments in Yuxiangyouzhan and Xiangyaxiangzhan. (**c**) Stomatal conductance under three temperature treatments in Yuxiangyouzhan and Xiangyaxiangzhan. (**d**) Transpiration rate under three temperature treatments in Yuxiangyouzhan and Xiangyaxiangzhan. LT represents low rhizosphere temperature (17.5 ± 1.5 °C); AT represents ambient rhizosphere temperature (28 ± 1.0 °C); HT represents high rhizosphere temperature (38.5 ± 1.5 °C). The vertical bars with different lowercase letters above are significantly different at *p* = 0.05 according to LSD tests. The capped bars represent the SDs.

No significant effect on the intercellular CO_2_ concentration, stomatal conductance, net photosynthetic rate or transpiration rate was detected in Yuxiangyouzhan. For Xiangyaxiangzhan, the low-temperature treatment caused a significant reduction in stomatal conductance and transpiration rate, while the high-temperature treatment resulted in a significant increase in stomatal conductance and transpiration rate. Additionally, the intercellular CO_2_ concentration and net photosynthetic rate were not significantly affected by any temperature treatment. Therefore, the Pn, Cond, and Tr were reduced significantly under low rhizosphere temperatures in the non-super rice, but they were not notably affected in the super rice. The high rhizosphere temperature had no effect on the Cond or Tr of the super rice, while a pronounced increase in Cond and Tr of the leaves of non-super rice was detected (Fig. 6).

### Correlation analysis

**Figure 7:**
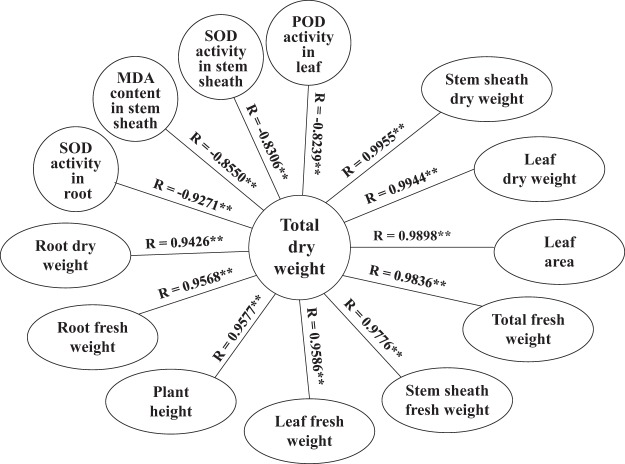
Correlation analyses of agronomic traits and antioxidant activity. R value represent correlation coefficient. **Significance at *p* < 0.01; ns represent non-significance.

The correlation analysis results, as depicted by correlation coefficients, revealed that the total dry weight exhibited a significantly positive correlation with the root dry weight (R = 0.9426**), stem sheath dry weight (R = 0.9955**), leaf dry weight (R = 0.9944**), root fresh weight (R = 0.9568**), stem sheath fresh weight (R = 0.9776**), leaf fresh weight (R = 0.9586**), total fresh weight (R = 0.9836**), plant height (R = 0.9577**) and leaf area (R = 0.9898**) but a negative correlation with SOD activity in the roots (R = −0.9271**), SOD activity in the stems (R = −0.8306**), POD activity in the leaves (R = −0.8239**) and MDA content in the stems (R = −0.8550**) (Fig. 7).

## Discussion

Significant effects of cultivar and temperature and their interaction on some plant growth parameters, such as total dry weight, leaf dry weight and total fresh weight, were observed (Table 1). Further, this study showed that high temperature resulted in a significant increase in total fresh weight and leaf fresh weight for super rice, while no effect was observed in any organ fresh weight for non-super rice (Table 3), which was in accordance with a report stating that the fresh weight and chlorophyll decreased significantly under high-temperature conditions (42 °C)^39^. Additionally, at low temperatures, a notable reduction was detected in total fresh weight, leaf fresh weight and leaf fresh weight per area for super rice, while in all organs, fresh weight (total fresh weight, root fresh weight, stem sheath weight and leaf fresh weight) were reduced significantly for non-super rice (Table 3). This finding was consistent with a report pointing out that low temperature (15 °C) and high temperature (above 30 °C) caused a decrease in seedling fresh weight^40^. The low temperature caused a significant reduction in leaf dry weight for super rice and total dry weight, stem sheath dry weight and leaf dry weight for non-super rice in this study. In addition, the high temperature resulted in a notable reduction in the total dry weight, stem sheath dry weight and leaf dry weight of super rice, but no effect on organ dry weight (total dry weight, root dry weight, stem sheath dry weight, leaf dry weight and leaf dry weight per area) for non-super rice was detected in this experiment (Table 2). This result was in accordance with what had been found when the temperature was below 12 °C—seedling height and dry weight were reduced^41^. The SPAD value and leave area were notably reduced at low temperatures for super rice and non-super rice in the low-temperature treatment in this study (Table 4). This result is different from a previous report in which the SPAD value was relatively high at low temperatures (16–25 °C). The reason for the difference may be the continuous treatment time^25^. In contrast, at high air temperatures (39 °C and 40 °C), the SPAD value and leaf area index decrease^42^, and high air temperature (29 °C) can result in a significant reduction in SPAD values^43^. In this study, the SPAD value and leaf area were not affected by super rice and non-super rice in the high-temperature treatment. In terms of the plant height of the super rice and non-super rice in the high-temperature treatment (Table 4), the results are different from those of a report in which high temperature (30 °C) caused an increase in the rate of plant development^19^; however, in this study for super rice, the plant height was not notably affected by low temperature (Table 4). In other words, the response of photosynthesis and plant development to temperature differs with species and among studies^44^. In addition, significant correlations between the total dry weight and some agronomic traits were assessed (Fig. 7).

Temperature, as one of the abiotic stresses, has a great influence on photosynthesis and photosynthetic parameters in plants^45–47^. In this study, the cultivar significantly affected the stomatal conductance, and the temperature treatment dramatically affected the stomatal conductance, net photosynthetic rate, and transpiration rate (Table 1). Photosynthesis is the key source contributing to the dry matter of rice grain^48^. Low temperature (15/10 °C) can cause a reduction in photosynthetic acclimation, can disrupt the balance of the absolute rates of RuBP regeneration and carboxylation, and can change the optimum temperature of RuBP carboxylation^49^. In this study, the non-super rice exhibited a significant increase in stomatal conductance and transpiration rate as the temperature increased (Fig. 6), which is consistent with previous studies in which the stomatal conductance increased with temperatures up to 35 °C^50^. The present study revealed no marked effect on the intercellular CO_2_ concentration, stomatal conductance, net photosynthetic rate, or transpiration rate in the temperature treatment for the super rice (Fig. 6), which was in accordance with a report showing that the net photosynthetic rate was not markedly different under high temperature (40 °C) compared with the control temperature (24 °C)^51^ and was consistent with a report in which there was no notable relation between photosynthesis and low temperature (16–25 °C)^25^.

The antioxidant enzyme activity was affected by cultivar, temperature and the interaction of cultivar and temperature, while it differed across plant tissues (Table 1). Antioxidant activity increases in response to heat^52^. Moreover, the SOD and POD activities in the stem sheaths and leaves remained higher in the low-temperature treatment than in the control temperature treatment in super rice. For the non-super rice, the low temperature caused a notable rise in SOD activity in the roots and in POD activity in the roots and stem sheaths, while a marked reduction in POD activity in the leaves occurred in non-super rice (Figs 1; 2). This result is consistent with a report in which low air temperature (8 °C) can cause a striking increase in SOD activity, CAT activity, and ascorbate peroxidase (APX) activity in rice cultivars that have chilling tolerance and a notable decrease in rice cultivars that are sensitive to chilling^53^. This experiment showed that at high temperature, SOD activity and POD activity in leaves significantly increased in super rice, and a notable increase in SOD activity in the roots and in POD activity in the roots and stem sheaths in non-super rice were detected (Figs 1, 2), which was in line with a report that heat stress (40 °C) can cause an increase in superoxide dismutase, guaiacol peroxidase, ascorbate peroxidase and glutathione reductase enzymes^54^. The activities of antioxidant enzymes (SOD activity, POD activity, CAT content, etc.) increased in response to heat treatment^55^. However, at high temperature, POD activity in the roots and stem sheaths was reduced significantly in super rice, and a striking reduction in SOD activity in the leaves and POD activity in the leaves of non-super rice was detected (Figs 1, 2). CAT activity in the stem sheaths of super rice and in the leaves of non-super rice was reduced significantly at low temperatures (Fig. 3), which is in accordance with a report that indicated that under chilling conditions (5 °C), CAT activities were drastically decreased in chilling-tolerant rice and chilling-sensitive rice^56^. In addition, this study suggested that the high temperature resulted in a decrease in CAT activity in the roots and stem sheaths of super rice and a significant reduction in CAT activity in the roots and leaves of non-super rice (Fig. 3), which were consistent with the finding that a reduction in CAT activity was found in response to heat stress (42 °C)^39^ and in accordance with a report that heat can result in a slight rise in CAT, APX, and GR activity during recovery from chilling^57^. Additionally, significant correlations between total dry weight and SOD activity in the stem sheaths and roots and POD activity in the leaves were detected (Fig. 7).

Membrane lipid peroxidation (MDA and proline contents) was found to be affected by cultivar and temperature and their interaction but varied across different plant tissues (Table 1). At low temperatures, the MDA content in the stem sheaths and leaves of super rice and in the leaves of non-super rice increased notably but decreased significantly in the roots of super rice. The roots and stem sheaths of non-super rice were not markedly affected in this experiment (Fig. 4). This resultpointed out that the chilling treatment caused a marked increase in MDA content^58^. The MDA content in the roots, stem sheaths and leaves was reduced at high temperatures in the super rice, but no marked difference was observed in the non-super rice (Fig. 4), which was different from the report showing that heat stress (42 °C) caused a significant increase in MDA content, hydrogen peroxide (H_2_O_2_) and proline content^39^, while high temperature (29 °C) caused an increase in MDA content^43^. At high temperatures, the proline content in the stem sheaths was reduced notably in super rice and increased significantly in super rice (Fig. 5). In addition, for non-super rice, the proline content in the leaves was increased in the low- and high-temperature treatments (Fig. 5). This result was consistent with a report showing that low temperature (15 °C) and high temperature (over 25 °C) caused a reduction in proline content^40^. Additionally, high-temperature (30–37 °C)-tolerant rice cultivars have relatively high chlorophyll, solutes, proline, and MDA contents in their flag leaf^38^. In this study, for non-super rice, the high temperature caused a rise in proline content in the roots and stem sheaths, while the low temperature also caused an increase in proline content in the stem sheaths (Fig. 5). Moreover, the total dry weight was significantly associated with MDA content in the stem sheaths.

**Figure 8:**
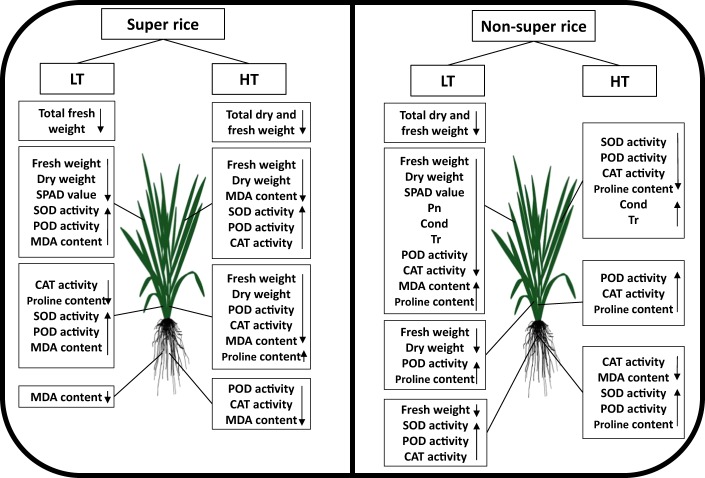
The response of super and non-super rice cultivars to high and low rhizosphere temperatures at the tillering stage.

Overall, the agronomic and physiological responses of the super and non-super rice cultivars to the rhizosphere temperature at the tillering stage are illustrated in Fig. 8. Under the low rhizosphere temperature, the super rice and non-super rice presented some similar parameters, e.g., total fresh weight, fresh weight of the leaves, dry weight of the leaves, SPAD values, MDA content in the leaves, POD activity in the stems and MDA content in the roots. Nevertheless, non-super rice appeared to present decreased POD activity in the leaves, fresh weight of the stems, dry weight of the stems and fresh weight of the roots, while super rice had no effect on the investigated parameters. Moreover, some parameters of super rice; e.g., total fresh and dry weight, fresh weight of the leaves, and fresh weight of the stems, declined at high rhizosphere temperatures, and these parameters were not affected in non-super rice. The results suggested that the damage caused by high temperature in super rice cultivars and low temperature in non-super rice cultivars was remarkable.

## Conclusion

Cultivar, rhizosphere temperature and their interaction had a marked effect on the total dry weight of super rice and non-super rice. The total dry weight of super rice was strongly affected by the high rhizosphere temperature and decreased by 16.26%, and the significant decline in total dry weight was due to the marked decreases in stem sheath dry weight and fresh weight, leaf dry weight and fresh weight, total fresh weight, POD activity in the roots and stems, CAT activity in the roots and stems, and MDA content. In addition, the total dry weight of the non-super rice was significantly influenced by low rhizosphere temperature and decreased by 41.06%, and the striking reduction in total dry weight was mainly due to the significant reduction in the stem sheath dry weight, leaf dry weight, plant fresh weight, SPAD values, plant height, leaf area, POD activity in the leaves, CAT activity in the leaves and proline content in the leaves. Moreover, correlations between the total dry weight and the investigated parameters were revealed. Additional studies to interpret the influence of rhizosphere temperatures on super rice and non-super rice at the molecular level are needed.

## Methods

### Experimentation and treatments

The two rice cultivars, the super rice cultivar ‘Yuxiangyouzhan’ and the non-super rice cultivar ‘Xiangyaxiangzhan’, are main commercial rice cultivars in South China and were used in this study. Yuxiangyouzhan, the super rice, is not sensitive to low temperatures during the seedling stage and has high seed yields, as well as other positive attributes^59,60^. Xiangyaxiangzhan, a non-super rice, can withstand increased temperatures (21/15 °C, 27/21 °C, 33 °C/27 °C), and its photosynthesis and synthesis of photosynthetic pigments are enhanced^61^; this cultivar is sensitive to low temperatures and has a low survival rate^62^. Moreover, Yuxiangyouzhan has a higher heat resistance than does Xiangyaxiangzhan^63^. A pot experiment was carried out from March to May in 2018 at the College of Agriculture, South China Agricultural University. Seedlings at the three-leaf stage were transplanted into pots (the bottom and upper diameter were 16 cm, and the height was 16 cm) filled with 2 L of Kimura nutrient solution (pH = 4.9), with four seedlings per pot and six replications for each treatment.

The temperature treatments were controlled by artificially adding water at different temperatures during the tillering stage. The treatments were continuous for two days, and the treatment period was from 9:00 a.m. until 12:00 p.m. every day. The three temperature treatments were as follows: (i) LT, low-temperature treatment (17.5 ± 1.5 °C); (ii) AT, ambient-temperature treatment (28 ± 1.0 °C), taken as the control; and (iii) HT, high-temperature treatment (38.5 ± 1.5 °C). The temperature treatments set up in this study were fully considered in previous studies^35^. A rhizosphere temperature higher than 37 °C significantly inhibits plant growth, and the impact depends on the duration of the treatments. For a short-term treatment experiment with low or high temperature, extremely low or high temperatures employed could achieve improved effects.

### Determination of fresh weight and dry weight

Ten representative seedlings were harvested at the end of the treatment and were separated into leaves, stem sheaths and roots. The samples were weighed after washing and being wiped dry for the determination of fresh weight. Then, the sample was oven-dried to a constant weight at 80 °C, and the dry weight was then measured.

### Determination of the SOD activity, POD activity, CAT activity, MDA content and proline content in the roots, stem sheaths and leaves

The representative seedlings were harvested and separated into roots, stem sheaths and leaves. They were then immediately put into liquid nitrogen for 1 minute and then stored at −80 °C until determination of the SOD activity, POD activity, CAT activity, MDA content and proline content in the roots, stem sheaths and leaves.

Crude enzymes were extracted by referring to methods of Lee and Lee^64^. In brief, 0.30 g of fresh samples was homogenized with 3 ml of 100 mM PBS solution, transferred to a centrifuge tube and then centrifuged at 12000 rpm at 4 °C for 15 min. The supernatant consisted of crude enzyme extract.

SOD activity was measured according to previous methods^65^. Briefly, the crude extract enzyme solution was added to a reaction solution containing 1.5 ml of 50 mM sodium phosphate buffer, 0.3 ml of 130 mM Met, 0.3 ml of 750 μM NBT, 0.3 ml of 100 μM EDTA-Na, and 0.3 ml of 20 μM riboflavin. The samples were placed under 4000 lx light for 20 minutes. The absorption value was recorded at 560 nm. The inhibition of 50% of NBT photochemical reduction was used to calculate the SOD activity. One unit of SOD activity was defined as the amount that caused a 50% reduction in the absorbance at 560 nm. The SOD activity was defined as units per gram of fresh weight (FW).

The POD activity was measured using a previously described procedure^66^. The crude extract enzyme solution (0.05 ml) was added to the reaction solution containing 1.0 ml of 50 mM PBS, 1.0 ml of 0.3% H_2_O_2_, and 0.95 ml of 0.2% guaiacol. The absorbance was determined at 470 nm, with 4 intervals and 30 seconds for each interval. A change in absorbance every minute by 0.01 was defined as one unit (U) of activity. The POD activity was defined as units per gram of fresh weight (FW).The CAT activity was evaluated by using a previously reported method^67^. The crude enzyme extract was added to the reaction solution containing 1.95 ml of ultrapure water and 1.0 ml of 0.3% H_2_O_2_. The absorbance was read at 240 nm every 30 seconds, with 4 replications. An absorbance change of 0.01 was defined as one unit (U) of CAT activity. The CAT activity was defined as units per gram of fresh weight (FW).

The MDA content was measured according to a previously described method^68^. First, 0.5% thiobarbituric acid (TBA) and the crude enzyme extract were mixed, cooled after heating in a boiling water bath for 30 minutes, and then centrifuged for 15 minutes (3000 rpm). The absorbance at 532 nm, 600 nm and 450 nm was then measured. Then, the MDA content in the different plant tissues was calculated.

The proline content was determined according to a previously described method^69^. Briefly, samples of 0.30 g were added to 3% sulfosalicylic acid, placed into boiling water for 10 min, and then cooled. After filtration, the extract and 2 ml of glacial acetic acid together with 3 ml of ninhydrin reagent were mixed, extracted with 4 ml of toluene, and then heated in a boiling water bath for 30 min. The mixture was then cooled in an ice bath for 20 min. The toluene extraction was subsequently centrifuged for 5 min (4000 rpm). The absorbance was then recorded at 520 nm. The proline content was expressed as micrograms per gram of fresh weight (FW).

### Determination of the photosynthetic rate, intercellular CO_2_ concentration, stomatal conductance and transpiration rate

The intercellular CO_2_ concentration, stomatal conductance, photosynthetic rate and transpiration rate of the leaves were detected by a portable photosynthesis system (LI-6400XT, Li-COR, Lincoln, NE, USA) from the top fully expanded leaf from 9:00–11:00 a.m., with five plants for each treatment, according a previously described method^70^.

### Statistics

The experimental data were analyzed by Statistix version 8 (Analytical Software, Tallahassee, Florida, USA). To determine the significance of the temperature treatments and cultivars, an analysis of variance was used; cultivar was the main factor, and temperature was the subfactor. The means between treatments were compared based on the least significant difference (LSD) test at the 0.05 probability level. Correlations between cultivars, temperature and their interaction were determined by Pearson’s analyses. Figures were plotted by Excel 2010.

## Data Availability

All data generated or analyzed during this study are included in the article.
